# A Micro Absolute Distance Measurement Method Based on Dispersion Compensated Polarized Low-Coherence Interferometry

**DOI:** 10.3390/s20041168

**Published:** 2020-02-20

**Authors:** Xun Sun, Kunpeng Feng, Jiwen Cui, Hong Dang, Yizhao Niu, Xuping Zhang

**Affiliations:** 1 Institute of Optical Communication Engineering, College of Engineering and Applied Sciences, Nanjing University, Nanjing 210093, China; sx2503962673@163.com (X.S.); xpzhang@nju.edu.cn (X.Z.); 2 Key Laboratory of Intelligent Optical Sensing and Manipulation (Nanjing University), Ministry of Education, Nanjing University, Nanjing 210093, China; 3 Center of Ultra-precision Optoelectronic Instrument, Harbin Institute of Technology, Harbin 150080, China; danghong@hit.edu.cn (H.D.); m18846819526@163.com (Y.N.); 4 Key Lab of Ultra-precision Intelligent Instrumentation, Harbin Institute of Technology, Ministry of Industry and Information Technology, Harbin 150080, China

**Keywords:** micro absolute distance measurement, low-coherence interferometry, Fabry-Perot sensors

## Abstract

Micro absolute distance measurement (MADM) is widely used in industrial and military fields. To achieve high accuracy and frequency response, a polarized low-coherence interferometry (PLCI)-based method for MADM is proposed. The nearly linear relationship between the envelope center and m-order PLCI fringe (PLCIF) peak center is found and verified. Dispersion compensation is achieved by fringe peak position estimation and polynomial fitting to get rid of the dependence on an a priori model and birefringence parameters, and make this method very robust. Meanwhile, the zero-order PLCIF center is estimated and located to demodulate the measured displacement. Then, the measurement accuracy is raised by polynomial fittings. In comparison to conventional methods, the proposed method can effectively avoid jump errors and has a higher accuracy. Experimental results indicate that the measurement accuracy is higher than 19.51 nm, the resolution is better than 2 nm, and its processing data rate can reach 35 kHz.

## 1. Introduction

Micro absolute distance measurement (MADM) is widely used in industrial and military fields, such as surface profile measurement, optical coherence tomography, and optical fiber sensing applications such as pressure in hash environment, refractive index, and thickness of transparent materials [1,2,3,4]. Absolute length interferometers are known as being highly specialist, capable of being operated only under strictly controlled environmental conditions and are also very expensive [5,6,7]. However, recent developments in low-coherence interferometry (LCI), in particular its convenience of relatively low cost, light weight and high response, have led to an upsurge in interest in this area. This is motivated by the possibility of bringing ultra-high precision absolute distance measurement, typically sub-micrometer, out of the standards laboratory and into wider industrial use [8,9,10].

LCI is considered as a most promising demodulation method as results of its high signal-to-noise-ratio (SNR) and compact optical integrated structure. Chang-Yun Lee reported in 2019 a scanned LCI for MADM to accomplish tilt metrology on rough dielectric surfaces and intensity-based peak method is utilized to demodulated scanning signal [11]. The deviation for the 5 repeated measurements is 0.05’. Chen proposed a LCI without scanning components and used two optical plates to form an optical wedge as a reference interferometry [12]. The MADM is achieved by intensity-based peak method. The nonlinearity is 0.67%. However, PCI is facing with processing difficulty can cost for MADM, which need an optical wedge of a very little wedge angle and thickness. Polarized LCI (PLCI) of LCI is considered as a most promising demodulation method as results of its low requirement of optical wedge. But austere challenges of dispersion caused by birefringence optical wedge (BOW) should be overcome to improve its measurement accuracy. Liu proposed in 2018 a PLCI system for MADM of a FP (Fabry-Perot) sensor [13]. A BOW of 1.5 mm in thickness and 4° in wedge angle is designed which could be easily manufactured by standard process. Conventional intensity-based peak or phase-based detection algorithms cannot be directly utilized in PLCI, which would lead to misidentification of the fringe order and cause jump errors [5,14]. Several researches have been done in this particular issue. In 2010, Lehmann proposed a PLCI based method for MADM, and used static tilted grating frequency domain optical delay line and depth-variant kernel to compensate dispersion [15]. This depth-dependent method is mainly based on a priori/posteriori knowledge and focuses on the envelope full width at half maximum (FWHM), not the envelope peak position. In 2013, Liu built a dispersion model of PLCI with a Gauss profile light source [1]. The fringe envelope center position and a position-dependent compensation are used to demodulate optical fiber Fabry-Perot (FP) sensors. The fringe envelope center position is sensitive to noise which would influence sensing accuracy. In 2015, Wang proposed a spatial-frequency domain analysis (SFDA) based method with dispersion comparison of wavenumber and built the nonlinear relationship between wavenumber and discrete Fourier transform (DFT) serial number [16]. This method retrieves the relative phase around a monochromatic wavenumber peak to achieve its absolute phase and measurement displacement. The arc tangent phase unwrapping accuracy of SFDA depends on the SNR and could induce misidentification of the fringe order. On the other hand, the existing PLCI demodulation methods for phase compensation are all based on an a priori model and birefringence parameters of BOWs. Due to the influence of light source profile and the undetermined birefringence parameters errors, the PLCI fringe (PLCIF) would be different from the estimated models and cause jump errors. So a robust PLCI demodulation method independent on model and birefringence parameters is highly demanded for MADM of a high accuracy.

In this paper, a MADM method based on PLCI without dependences on the priori model and birefringence parameters is proposed to avoid jump errors and make it robust. A new approximate PLCI model is first built and investigated with light sources of different profiles. Then, a nearly liner relationship between PLCIF peak and envelope center is both theoretically derived and experimentally verified. In this method, the position of a certain order PLCIF can be estimated and its peak position can be utilized to demodulate the measurement displacement. A polynomial fitting of the measured peak position and displacement is accomplished to compensate dispersion and raise the sensing accuracy. Finally, an extrinsic FP sensor constructed by an optical fiber and a moving mirror is utilized to evaluate the proposed method. Experimental results indicate that this method has robust dispersion compensation ability and can effectively avoid jump errors. The MADM accuracy is higher than 19.5 nm, the resolution is better than 2 nm within a measurement range of ~5 μm, and signal processing time on an embedded system is ~22 μs and its processing data rate can reach 35 kHz. The repeatability at different measured distance is better than ~8.39 nm, the stability within 30 min is better than ~26.76 nm and the proposed method has a low harmonic distortion of ~25 dB at the first harmonic frequency under a sinusoidal wave displacement signal of 1 kHz.

In Section 2, the principle of PLCI for MADM is presented. Then its model is built and simulated. The proposed method is also demonstrated in detailed. In Section 3, the proposed method is experimentally tested and compared with conventional methods. Finally, conclusions are provided in Section 4.

## 2. Principle of PLCI for MADM and the Proposed Method

In this section, first the basic principle of PLCI for MADM is demonstrated. Then, the nearly linear relationship between the envelope center and *m*-order peak center is found when the light source is in Gauss profile. Therewith, this relationship is also verified when the light source is in other uniform and measured profiles. Finally, discussion and summary is given to explain how to implement the proposed method in PLCI.

### 2.1. Basic Principle and Model of PLCI

**Figure 1:**
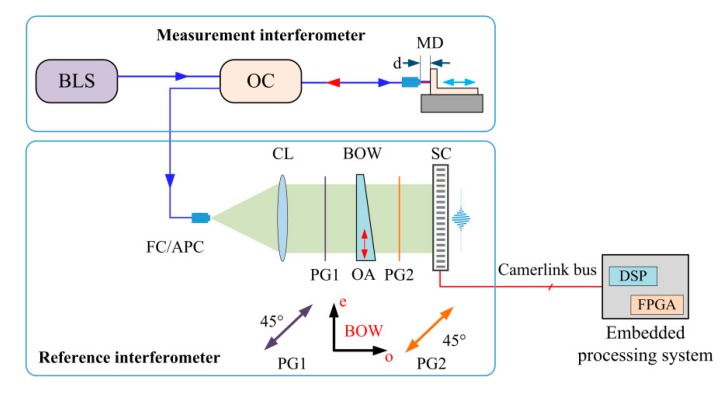
Schematic diagram of measurement and reference interferometer of a typical PLCI.

A typical PLCI configuration for MADM is illustrated in Figure 1. It consists of a measurement interferometer (MI) and a reference interferometer (RI). MI measures the absolute displacement and demodulates the measurement distance (MD) into spectrum variation. RI matches its spectrum with the spectrum of MI and generates PLCIF. MI consists of a broadband light source (BLS), an optical circulator (OC) and a device generating MD. RI consists of a collimation lens (CL), two orthorhombic polarization generators (PG), a BOW and a sampling camera (SC). The birefringence of BOW could generate two referencing light of different optical path differences.

In MI, the reflectivity of the interface between air and silica and equivalent reflectivity of the mirror interface and fiber coupling ratio is quite low. So MI can be considered as a two-beams-interference. In RI, two orthorhombic polarized optical beams occur interference, and Ring also is a two-beams-interference. Then, PLCIF on the SC can be expressed as a product integral of intensity transfer functions (TF) of MI, RI and BLS [11]:

(1) $$I(d,h)=\int_{-\infty }^{\infty }F_{\mathrm{MI}}(d,k)F_{\mathrm{RI}}(h,k)F_{s}(k)dk$$

where, *d* is MD, *k* = 2π/*λ* is wavenumber and *h* is the local thickness of BOW. *F*_MI_(*d*,*k*), *F*_RI_(*h*,*k*) and *F*_s_(*k*) are respectively the intensity TF of MI, RI and BLS.

MI is a multi-reflection interferometer. It contains two reflective interfaces, the reflectivity of the interface between air and silica and equivalent reflectivity of the mirror interface and fiber coupling ratio. Due to their reflectivity is lower than 0.05, the multi-reflection interferometer can be equivalent to a model of two beam interference, and the intensity of interference can be written as:

(2) $$I_{\mathrm{MI}}(d,k)={\left|\sqrt{P_{\mathrm{in}}}\sqrt{R_{1}}+\sqrt{P_{\mathrm{in}}}\sqrt{1-R_{1}}\sqrt{R_{2}}\exp(-2jkd)\right|}^{2}$$

where, *R*_1_ is the reflectivity of the interface between air and silica wherein *R*_1_ ≈ 0.04, *R*_2_ is the equivalent reflectivity of the mirror interface and fiber coupling ratio wherein *R*_2_ ≈ 0.05 and *P*_in_ is the intensity of the incident light.

Due to the reflectivities of the two interfaces in MI are very low, so the TF of MI can be simplified as below [1,9]:

(3) $$F_{MI}(d,k)=R_{1}+R_{2}-2\sqrt{R_{1}R_{2}}\cos(2kd)$$

BOW has ordinary and extraordinary axes of different optical velocity. So polarizations passing PG1 is equal in ordinary and extraordinary axis can be considered as two beams and their interference occurs when passing PG2. RI is an interference of two orthorhombic polarized optical beams, and its TF can be written as [1,9]:

(4) $$F_{RI}(h,k)=0.5\left\{1+\cos[khn(k)]\right\}$$

where, *n*(*k*) is the birefringence of BOW which varies with wavenumber and causes dispersion.

By substituting Equations (3) and (4) into Equation (1) and ignoring the nearly zero terms within thickness range of BOW, the integral express can be simplified into [1,9]:

(5) $$I(d,h)=\int_{-\infty }^{\infty }F_{s}(k)\left\{R_{1}+R_{2}-\sqrt{R_{1}R_{2}}\cos k[hn(k)-2d]\right\}dk$$

Once the TF of BLS is determined, the PLCIF on the SC can be achieved. The center of PLCIF would shift with the MD. Therefore, MADM can be demodulated using the PLCIF. Then, the relationship between PLCIF and MD is investigated by running simulations with Equation (5).

### 2.2. PLCI for MADM with a BLS of Gauss Profile

**Figure 2:**
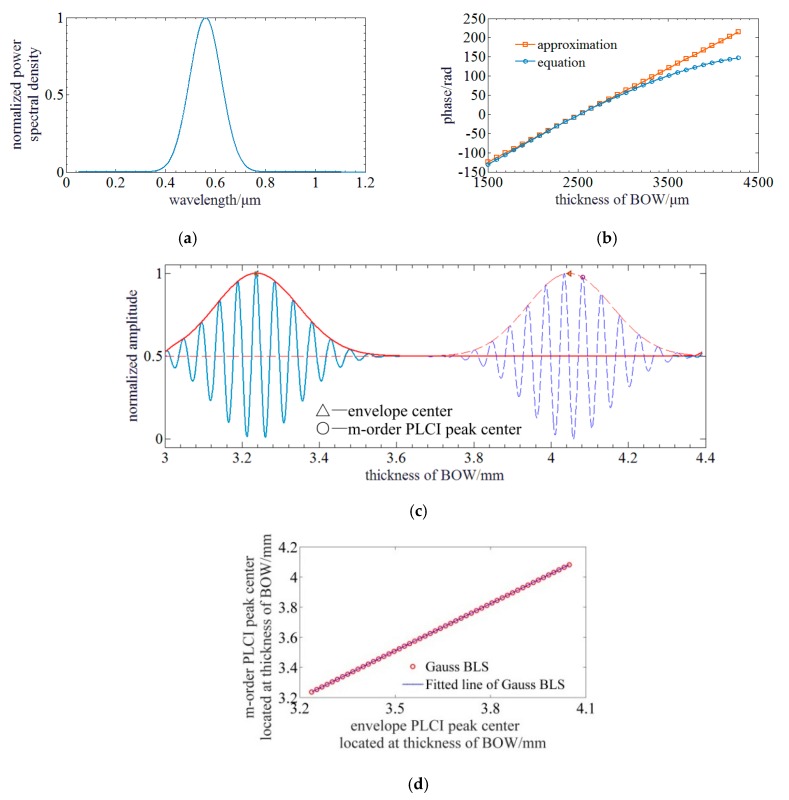
Simulation of PLCI for MADM with a BLS of Gauss profile: (**a**) The profile of the Gauss BLS; (**b**) The phase curves of equation and approximation; (**c**) PLCIF of BLS in Gauss: The solid and dotted lines represent the PLCIFs at different MDs. The blue and red lines represent the measured PLCIFs achieved by SC and envelopes curves achieved by FFT method; (**d**) Relationship between envelope and *m*-order PLCIF peak center.

When the BLS is in Gauss profile as illustrated in Figure 2a, $F_{s}(k)$ can be written as:

(6) $$F_{s}(k)=I_{o}\exp\left(-{\left[\alpha \left(k-k_{0}\right)\right]}^{2}\right)$$

where, $\alpha =2\sqrt{\ln\left(2\right)}/\Delta k$. *I*_0_, *k*_0_ and Δ*k* are the power spectral density, central wavenumber and bandwidth of the BLS.

By substituting Equation (6) into Equation (5), PLCIF of a BLS in Gauss profile can be written as [1]:

(7) $$\begin{array}{l} I(d,h)=\sqrt{R_{1}R_{2}}{\left(1+\eta^{2}\right)}^{-1/4}\exp\left[-\frac{{\left(z\Delta k\right)}^{2}}{4\gamma (1+\eta^{2})}\right]\\ \;\times \cos\left[zk_{0}-\beta k_{0}^{2}d+0.5\arctan\eta -\frac{\eta \Delta k^{2}z^{2}}{4\gamma (1+\eta^{2})}\right]+C \end{array}$$

where, $z=(n_{0}+\beta k_{0})h-2d$, $\eta =\beta \Delta k^{2}h/\gamma$, γ=4ln2, $\beta =n^{\prime }\left(k_{0}\right)$ is the derivative of birefringence at *k*_0_, *n*_0_ is the birefringence of BOW at the central wavelength of BLS, and *C* is an integration constant.

The cosine term in Equation (5) can be simplified by Taylor expansion around the center wavenumber *k*_0_:

(8) $$\begin{array}{l} k[hn(k)-2d]\approx h\left\{k_{0}n\left(k_{0}\right)+\left[n\left(k_{0}\right)+k_{0}n^{\prime }\left(k_{0}\right)\right]\left(k-k_{0}\right)+o\left[{\left(k-k_{0}\right)}^{2}\right]\right\}-2kd\\ \;=k\left\{\left[k_{0}n^{\prime }\left(k_{0}\right)+n\left(k_{0}\right)\right]h-2d\right\}-k_{0}^{2}n^{\prime }\left(k_{0}\right)h\\ \;=k\left[(k_{0}\beta +n_{0})h-2d\right]-k_{0}^{2}\beta h \end{array}$$

where, *n*(*k*) is the birefringence of BOW, $\beta =n^{\prime }\left(k_{0}\right)$ is the derivative of birefringence at *k*_0_, and *n*_0_ is the birefringence of BOW at the central wavelength of BLS.

Thuso, Equation (5) can be simplified using the Taylor expansion of cosine term. By substituting Equations (6) and (8) into Equation (5), PLCIF of a BLS in Gauss profile can be written as:

(9) $$I(d,h)=\frac{R\sqrt{\pi }}{\alpha }\cdot \left\{1-\exp\left[-\frac{{\left(z\Delta k\right)}^{2}}{\gamma }\right]\cdot \cos\left[n_{0}k_{0}h-2k_{0}d\right]\right\}$$

Simulation is run with following configurations. The central wavelength and FWHM of the BLS in Gauss profile are 560 nm and 150 nm. The corresponding wavenumber *k*_0_ is 0.01122/nm. The thickness of an MgF_2_ BOW located in the monitored range of a camera is 3 mm and 4.4 mm. The angle of this BOW is ~5.51°. The birefringence *n*_0_ and its derivative *β* of BOW at the central wavelength of BLS are respectively ~0.01184 and ~4.703 × 10^−5^. The SC has 2,048 pixels and its pixels pitch is 7.04 μm.

Figure 2b illiterates the results of phase curves by Equation (7) and approximation result of Equation (9). It indicates that the phase curves of both equation derivation and approximation are close to each other near the zero phase point or the envelope peak of PLCIF. However, they vary difference with the increment of the thickness of BOW or phase. The result achieved by Equation (7) gets a larger nonlinearity with the increment of the thickness of BOW.

Due to the dispersion of BOW, the center of envelope and the *m*-order PLCI peak would be not overlapped anymore. By Equation (9), the envelope center *h_c_* can be derived when the exponential term is zero, and the *m*-order PLCI peak *h_m_* can be derived when the cosine term is 2*m*π. So, *h_c_* and *h_m_* can be expressed as follows:

(10) $$\left\{ \begin{array}{l} h_{c}=2d/\left(k_{0}\beta +n_{0}\right)\\ h_{m}=\left(2k_{0}d+2m\pi \right)/\left(n_{0}k_{0}\right) \end{array}\right.$$

where, *m* is an integer.

Based on Equation (10), the micro absolute distance can be determined by measuring the envelope center *h_c_*. However, envelope center should be reconstructed by fast Fourier transform (FFT) method, and it cannot be directly detected which makes this demodulation method more sensitive to noise [16]. To achieve a high accurate MADM, the *m*-order PLCIF peak center is located to obtain the micro absolute distance. Nevertheless, it shifts with MD which causes misidentification of the fringe order and jump errors, so the location of the envelope center is first determined to estimate the *m*-order PLCIF peak center by Equation (10). Next, a more accurate quadratic fitting is conducted to achieve the location of the *m*-order PLCIF peak center. By substituting *h_c_* into *h_m_*, the relationship between envelope and *m*-order PLCIF peak centers can be expressed as a linear equation:

(11) $$h_{m}=ph_{c}+q$$

where, $p=\left(k_{0}\beta +n_{0}\right)/n_{0}$, $q=2m\pi /n_{0}k_{0}$. As a result of the nearly linear relationship between the envelope center and m-order peak center, no a priori model or input birefringence parameters are used to demodulate MADM. Based on Equation (11), the linear function parameters *p* and *q* can be obtained through an experimental test. Besides, the zero-order PLCIF peak is utilized to do the demodulation as a result of that a higher order PLCIF peak center may have the risk of exceeding the range of BOW. Although *h_m_* shifts with *h_c_*, it can be estimated by the measured envelope center and Equation (11). Figure 2d illustrates that they have a good linear relationship and *p* = 1.0436, *q* = –0.1424 and the linear fitting residual is ~0.093 mm. The quadratic polynomial fitting is used to locate the accurate center of the zero-order PLCIF peak $h_{m}{}^{\prime }$ within the PLCIF range centered by the estimated zero-order PLCIF peak *h_c_*, so the demodulated MD $d{}^{\prime }$ can be expressed as:

(12) $$d{}^{\prime }=n_{0}h_{m}{}^{\prime }/2$$

Due to the dispersion of BOW and the model error induced by linear approximation and Taylor expansion of Equation (8), the demodulated MD is not directly proportional to the actual MD which would influence the measurement accuracy.

**Figure 3:**
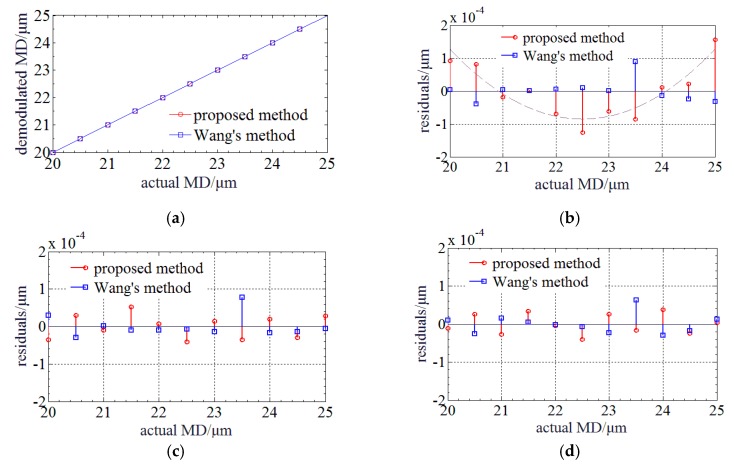
Relationship and fitting residuals between the demodulated and actual MD when BLS is in Gauss profile: (**a**) Relationship between the demodulated and actual MD; (**b**) Linear polynomial fitting residuals; (**c**) Quadratic polynomial fitting residuals; (**d**) Cubic polynomial fitting residuals.

Figure 3a illustrates the simulation results of both demodulated MDs achieved by the proposed and Wang’s method based on an a priori model [16]. It shows that they are nearly proportion to the actual MDs, and have no jump errors. Figure 3b shows that the maximum absolute residual (MAR) of the linear polynomial fitting by the proposed method is ~1.56 × 10^−4^ μm and worse than ~8.82 × 10^−5^ μm achieved by Wang’s method. Then, studies were done to improve the accuracy. The approximate relationship of the linear polynomial fitting residual between quadratic curves is found. It indicates that the demodulated and actual MDs satisfy a higher order polynomial. Figure 3c shows that the MARs of the quadratic polynomial fitting are respectively decreased to ~5.26 × 10^−5^ μm and ~7.78 × 10^−5^ μm. In comparison to Wang’s method, the proposed method can raise the accuracy by ~32.4%. Figure 3d shows that the MARs of the cubic polynomial fitting are respectively decreased to ~3.75 × 10^−5^ μm and ~6.33 × 10^−5^ μm. The accuracy is further raised by ~28.7% by cubic polynomial fitting. The order of polynomial fitting can be optimized based on the experimental results. Therefore, a dispersion compensation method for MADM based on PLCI is proposed. During the demodulation of micro absolute distance, the envelope center is first determined and then the zero-order PLCIF peak center is estimated. Next, the center of the zero-order PLCIF peak is accurately determined by centroid method or quadratic polynomial fitting (quadratic polynomial fitting is utilized in simulation), and MD can be calculated. Finally, the MD is compensated using polynomial fitting to reduce the influence of BOW’s dispersion.

### 2.3. PLCI for MADM with a BLS of Non-Gauss Profile

Next, this method is discussed with a BLS of non-Gauss profile. Based on the derivation of Equation (9), PLCIF of a BLS in typical square and triangular profiles can be written as:

(13) $$\left\{ \begin{array}{l} I_{\mathrm{square}}(d,h)=\frac{2R\sin(\Delta kz/2)}{z}\cos\left[k_{0}z-hk_{0}^{2}\beta \right]\\ I_{\mathrm{triangular}}(d,h)=\frac{2R}{z}\{\left[1-\cos(\Delta kz/2)\right]\left[\frac{1}{z}\cos(k_{0}z-hk_{0}^{2}\beta )-\frac{1}{\Delta k}-1\right]+\\ \;(\frac{1}{\Delta k}+1)\sin(\Delta kz/2)\cos\left[k_{0}z-hk_{0}^{2}\beta \right]\} \end{array}\right.$$

**Figure 4:**
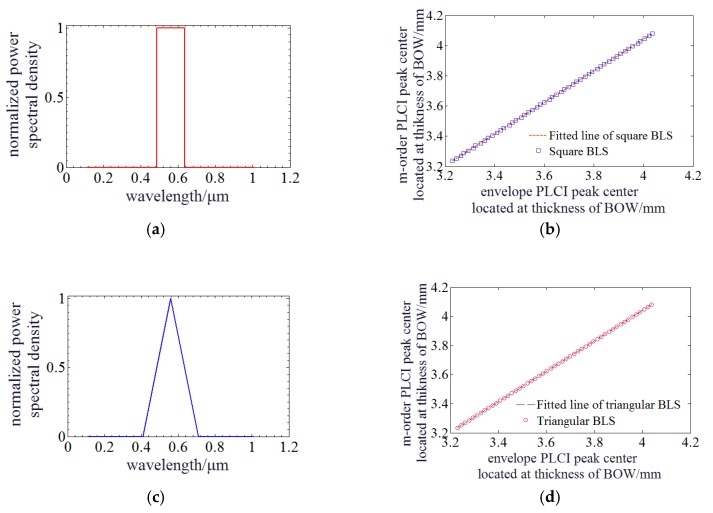
Simulation of PLCI for MADM with a BLS of square and triangular profile: (**a**) The profile of the square BLS; (**b**) Relationship between envelope and zero-order PLCIF peak center when BLS is in square profile; (**c**) The profile of the triangular BLS; (**d**) Relationship between envelope and zero-order PLCIF peak center when BLS is in triangular profile.

The expressions of PLCIF with a BLS in typical square and triangular profiles indicate that the relationship between *h_c_* and *h_m_* is not analytical. However, it also implies that *h_c_* is related to the expression of *z*, and *h_m_* is related to expression for $k_{0}z-hk_{0}^{2}\beta$. They are not overlapped due to dispersion. So simulation is run to investigate their relationship. Figure 4a,c illustrate BLS of typical square and triangular profiles and they are utilized to run the simulation. Figure 4b,d indicate that the linear relationship between envelope and zero-order PLCIF peak center still exists. The function parameters are *p* = 1.0457 and *q* = -0.1416 for BLS of square profile, and *p* = 1.0461 and *q* = 1.0461 for BLS of triangular profile. Their linear fitting residuals are ~0.126 mm and ~0.101 mm, respectively, so the proposed method is also available when BLS is in square and triangular profiles.

**Figure 5:**
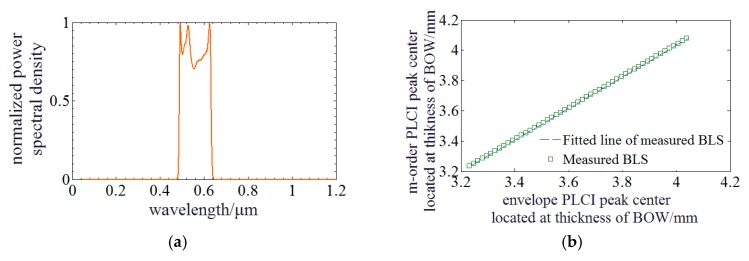

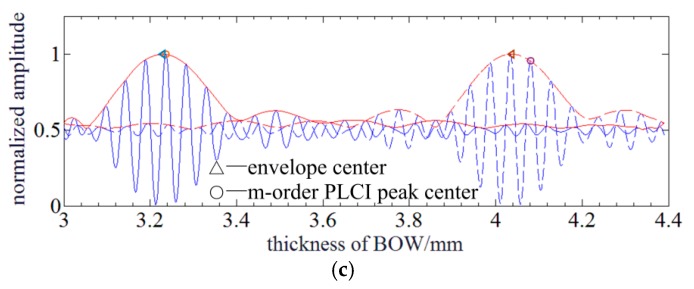
Simulation of PLCI for MADM with a BLS of measured profile: (**a**) The profile of the Gauss BLS; (**b**) Relationship between envelope and zero-order PLCIF peak center; (**c**) PLCIF of BLS in measured profile: The solid and dotted lines represent the PLCIFs at different MDs. The blue and red lines represent the measured PLCIFs achieved by SC and envelopes curves achieved by FFT method.

The zero-order PLCIF peak center can be determined by this linear relationship and then polynomial fitting is conducted to improve its sensing accuracy. Next, this method is discussed with a BLS of non-uniform profile. The BLS is a supercontinuum source SC-PRO-M produced by YSL Photonics (Wuhan, China). It has a broadband spectrum ranging from 400 nm to 2400 nm. Lowpass and highpass optical filters are utilized to achieve a broadband spectrum centered at 560 nm of a ~150 nm bandwidth. Its spectrum is measured as illustrated in Figure 5a. Then, this BLS of non-Gauss profile is used to run the simulation and verified the availability of the proposed method. Figure 5b,c illustrate that the zero-order PLCIF peak center shifts with the envelope center and they also represent a good linear relationship. The function parameters are *p* = 1.0450 and *q* = −0.1380 which is slightly different from these of BLS in Gauss profile. The linear fitting residuals are ~0.141 mm.

**Figure 6:**
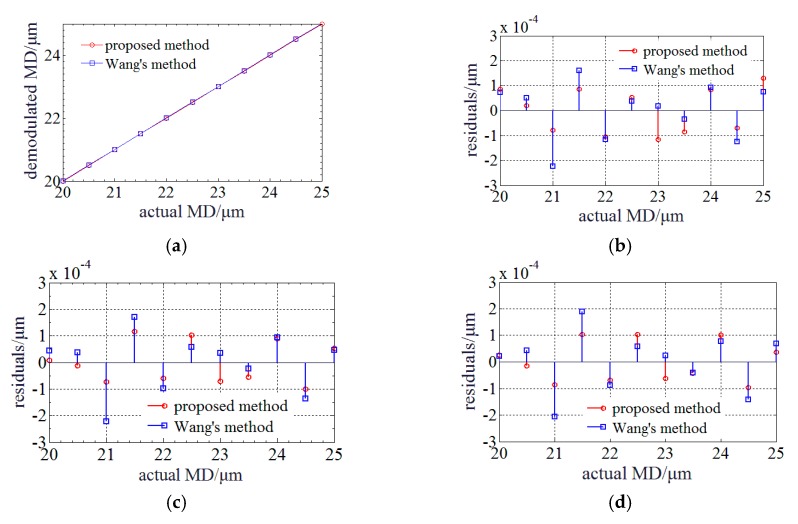
Relationship and fitting residuals between the demodulated and actual MD when BLS is in measured profile: (**a**) Relationship between the demodulated and actual MD; (**b**) Linear polynomial fitting residuals; (**c**) Quadratic polynomial fitting residuals; (**d**) Cubic polynomial fitting residuals.

Then, the measurement accuracy is verified by simulation. Figure 6a illustrates the relationship between the demodulated and actual MD of both demodulated MDs achieved by the proposed and Wang’s method based on an a priori model [16]. Figure 3b shows the result of the linear polynomial fitting residual and it indicates that MAR by the proposed method is ~1.17 × 10^−4^ μm and better than ~2.25 × 10^−5^ μm achieved by Wang’s method. The linear polynomial fitting residual is randomly distributed around zero which is different from that obeys a quadratic polynomial function when the BLS is in Gauss profile. Next, quadratic and cubic polynomial fitting is accomplished. The MARs of the proposed method are improved to 1.04 × 10^−4^ μm and 1.01 × 10^−4^ μm, respectively. The MARs of the Wang’s method are improved to 2.23 × 10^−4^ μm and 2.06 × 10^−4^ μm, respectively. The improvement of high order polynomial fitting is not significant. Therefore, the linear relationship between envelope and zero-order PLCIF peak center with measured profile BLS is verified and MADM can be accomplished.

The nearly linear relationship between the envelope center and *m*-order peak center when the BLS is in different profiles is summarized in Table 1. It can be seen that they have a good linear relationship but the fitting parameters are slightly different between each other.

In this section, the linear relationship between envelope and zero-order PLCIF peak center with non-Gauss profile BLS is verified by simulation. The measurement accuracy of the proposed method is ~50% higher than Wang’s method. Therefore, the proposed method is still available with a non-Gauss BLS in measured profile.

### 2.4. Summary of the Proposed MADM Method and Discussion

**Figure 7:**
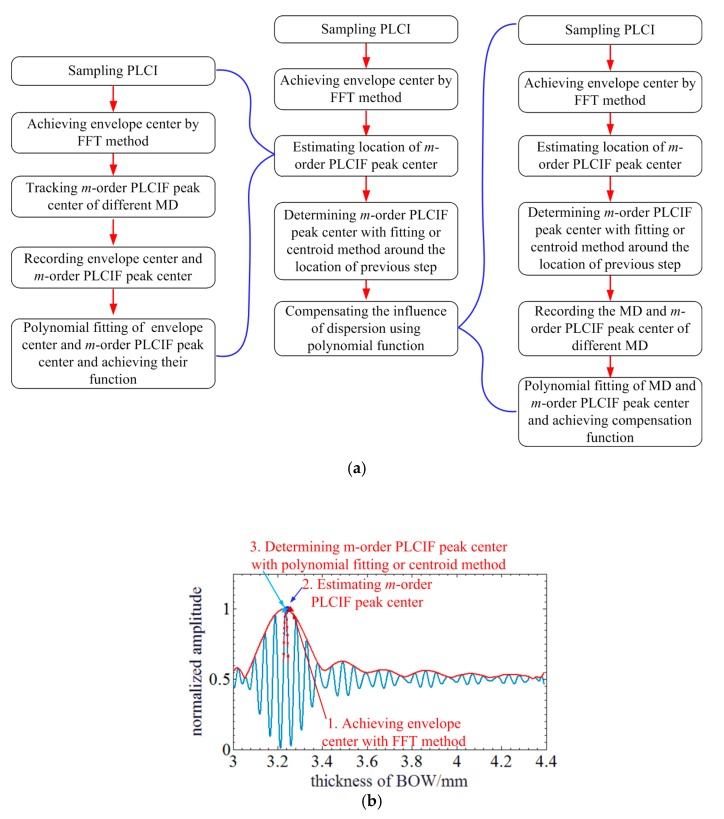
Summary of the proposed MADM method: (**a**) Processing flow of the proposed MADM method; (**b**) Schematic of the PLCIF processing flow.

Basing on the theoretical derivation and simulation of the previous sections, the processing flow of the proposed MADM method is illustrated in Figure 7a. The schematic of the PLCIF processing flow is also shown in Figure 7b. SC samples the PLCIF of PLCI for MADM. So, the envelope center of the PLCIF can be achieved by FFT method [16]. Then, the zero-order PLCIF peak center is estimated using its function of the envelope center. Next, the center of the zero-order PLCIF peak is accurately determined by centroid method or quadratic polynomial fitting around the point achieved by previous step. Finally, the MD is compensated using polynomial fitting to reduce the influence of BOW’s dispersion.

During the processing the proposed MADM method, two key system functions should be achieved through calibration. One is the function of zero-order PLCIF peak and envelope center. The other is the function for dispersion compensation.

The former can be achieved by following steps: (1) Sampling the PLCIF of PLCI for MADM; (2) Achieving envelope center by FFT method; (3) Tracking the zero-order PLCIF peak center; (4) Recording the envelope and zero-order PLCIF peak center of different MD; (5) Polynomial fitting these data points and achieving the function of zero-order PLCIF peak and envelope center.

The other can be achieved by following steps: (1) Sampling the PLCIF of PLCI for MADM; (2) Achieving envelope center by FFT method; (3) Estimating the location of zero-order PLCIF peak center; (4) Determining the center of the zero-order PLCIF peak by centroid method or quadratic polynomial fitting around the point achieved by previous step. (5) Recording the MADM and zero-order PLCIF peak center of different MD; (6) Polynomial fitting these data points and achieving the function for dispersion compensation.

Then, robust performance of the proposed method is discussed. The identification accuracy of the same order fringe during the demodulation can represent the robust performance of the proposed method. *h_c_* determined by a FFT based method [16] may consist of the error *h_cξ_* induced by noise. The error of estimated *h_m_* can be written as:

(14) $$h_{m}{}_{\xi }=ph_{c_{\xi }}$$

When the *h_mξ_* is less than ±T/2, misidentification of fringe order and jump error can be avoided. *T* is the period of the PLCIF and it can be expressed as:

(15) $$T=2\pi /\left(n_{0}k_{0}\right)$$

By substituting parameters of the BOW, *T* is 45.137 μm and the error *h_cξ_* is in an acceptable PLCIF error range of ~± 23.6 μm or 1.62% full scale of the thickness difference 1.4 mm of BOW. It indicates that this method can enhance the identification accuracy of the monitored PLCIF peak in this error band. The proposed method is more robust than the method by directly detecting envelope center.

## 3. Experiments and Results

### 3.1. Experimental Setups

**Figure 8:**
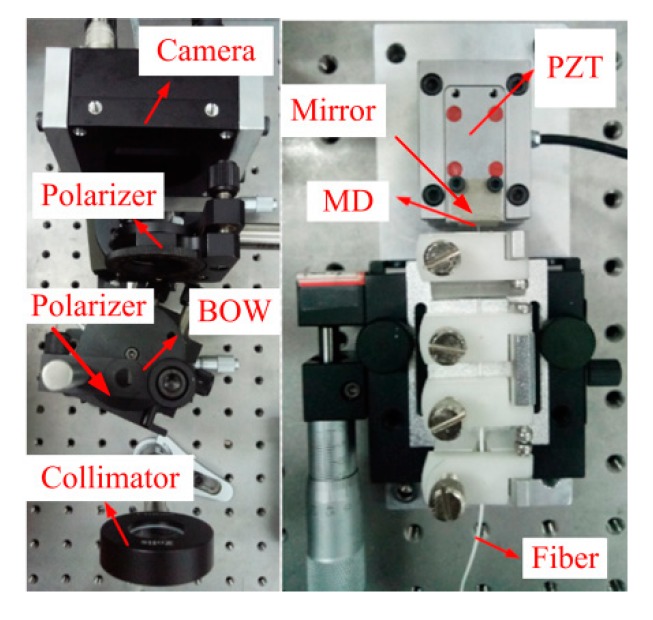
Experimental configuration.

Experiments are conducted to test the performance of the proposed method and experimental configuration is illustrated in Figure 8.

An LA-CM-02K-08A linear array camera produced by DALSA (Waterloo, Canada) is utilized to acquire PLCIF. It has same parameters with simulation. A P-753.1CD piezoelectric (PZT) actuator produced by PI (Karlsruhe, Germany) is utilized to generate accurate MD. Its travel range, resolution and resonant frequency are respectively 15 μm, 0.1 nm and 1.0 kHz. An Aluminum reflector is installed on the PZT actuator, so an extrinsic FP is formed with the mirror and the end of optical fiber. The cavity length of this FP sensor is the MD. The declared parameters of an MgF_2_ BOW are same with these in simulation and a polarizer is bonded on its front surface. An AC508-150-B-ML collimator produced by Thorlabs (Newton, NJ, USA) is utilized to collimate the beam from optical fiber. Its diameter and focus length are 50.87 mm and 150 mm. The BLS in experiment is a supercontinuum source SC-PRO-M produced by YSL Photonics. It has a broadband spectrum ranging from 400 nm to 2400 nm. Lowpass and highpass optical filters are utilized to achieve a broadband spectrum centered at 560 nm of a ~150 nm bandwidth.

### 3.2. Process of PLCIF and Achievement of the Key System Functions

**Figure 9:**
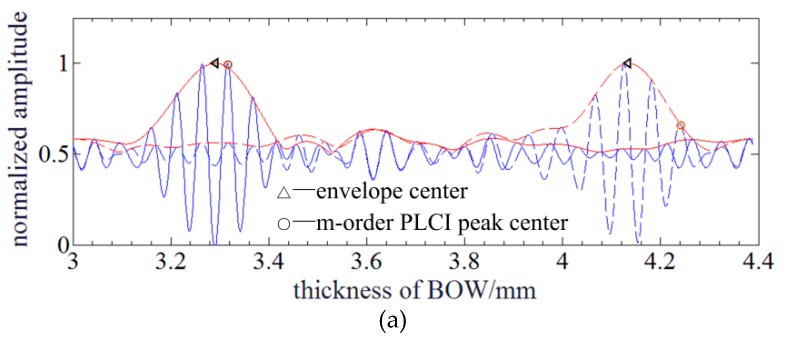

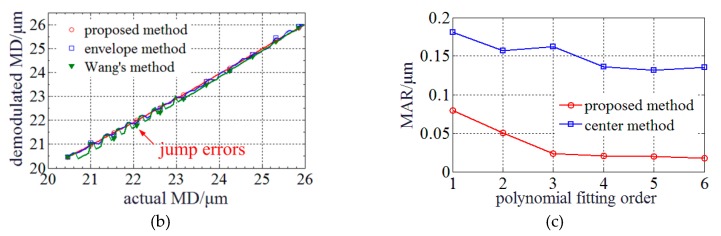
Experimental results of PLCIF and polynomial fittings: (**a**) PLCIF results when MDs are 20.4 μm and 26.0 μm: The blue and red lines represent the measured PLCIFs achieved by SC and envelopes curves achieved by FFT method; (**b**) Relationship between the demodulated and actual MDs; (**c**) MARs of different polynomial fitting orders.

Figure 9a shows that PLCIFs sampled by SC when MDs are 20.4 μm and 26.0 μm. The envelope center is achieved by FFT method [16] and the envelope is also illustrated. It can be seen that PLCIF and envelope is not completely symmetrical as results of the non-uniform distribution of the BLS intensity on the camera and stray lights. The location of the zero-order PLCIF center is tracked and recorded with different MD. Then, linear fitting based on Equation (10) is conducted and *p* and *q* can be determined as 1.0881 and 0.2648, respectively. So, the function of zero-order PLCIF peak and envelope center is obtained.

Then, PZT actuator executes an increment step of 60 nm and the MDs from 20.4 μm to 26.0 μm are demodulated in Figure 9b. It indicates that the demodulated MD achieved by the proposed method is smooth with no jump errors and neatly proportion to the actual MD. Directly detecting the envelope center also has no jump errors but has a worse linearity. Wang’s method is based on priori model and birefringence parameters and errors of model input parameters would cause phase unwrapping error, so it has many jump error points. The MARs of three methods are compared in Figure 9c. The MAR of the proposed method is much lower and quickly decreases when the fitting order increases from linear to cubic. However, the enhancement is not significant when the fitting order is higher than three, so a cubic polynomial fitting is utilized to achieve the function for dispersion compensation. In comparison to envelope center method, the measurement accuracy is ~19.5 nm with a cubic polynomial fitting and can be raised by 6.88 times.

### 3.3. Experimental Performance Tests

**Figure 10:**
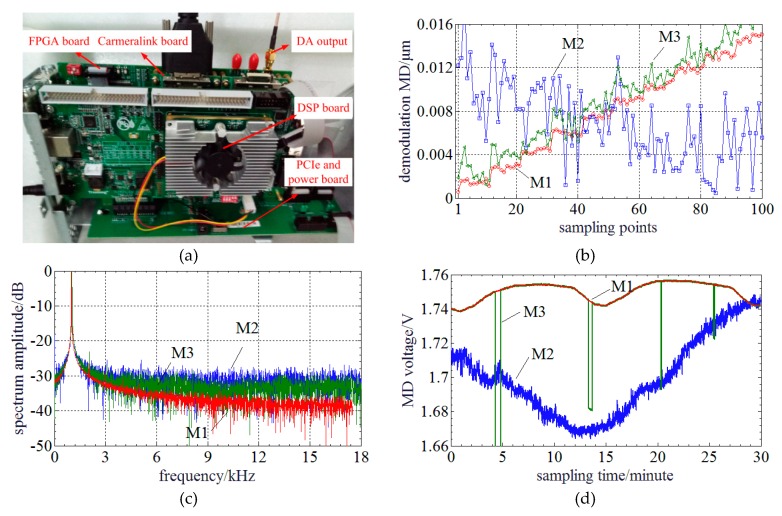
Experimental results of performance tests: (**a**) Photography of the embedded processing system; (**b**) Resolution test; (**c**) Harmonic spectrum of the frequency response test; (**c**) Harmonic spectrum of the frequency response test; (**d**) Stability test within 30 min (M1, M2 and M3 respectively represents the proposed method, envelope method and Wang’s method).

During the experimental performance tests, an embedded processing system is used to sample PLCIF image and run the proposed method. Figure 10a shows a photograph of this embedded processing system. It consists of a FPGA board, a DSP board, a CameraLink board and a PCIe/power board. The FPGA board is a Xilinx A7 (Xilinx, San Jose, CA, USA). It takes charge of sampling PLCIF image into inner FIFO register and executes FFT of the PLCIF image in a parallel pipelined architecture. The results of FFT are transferred into DSP board through 1× PCIe interface. The envelope center, zero-order PLCI peak center, dispersion compensation and MD are accomplished in the DSP board (C6657 dual core DSP, Texas Instruments, Dallas, TX, USA). The MD is converted into 0.5~3.3 V voltage and exported through a high speed DA. The full scale is 20 μm~25 μm and the displacement sensitivity is 0.56 V/μm. The PCIe/power board connects the FPGA board and DSP board with PCIe interface and powers these two boards. The signal processing time of the proposed method on this embedded system comprised of the Xilinx A7 FPGA and Texas Instruments C6657 dual core DSP is ~22 μs. Considering the DA configuration and data interaction process, the processing frequency of the MADM can reach 35 kHz.

The resolution of MADM is tested first. Figure 10b illustrates the results. It indicates that the proposed method can clearly distinguish MD step of 2 nm (~0.36‰ of a full scale) and has a less standard deviation Wang’s method. The experimental result of envelope method cannot distinguish the MD step.

Then, the frequency and harmonic response is experimentally tested. Limited to the PZT actuator, only frequency response of 1 kHz is tested. The amplitude of the vibration signal is 0.75 μm around ~22.3 μm. The sampling time window is 140 ms and sampling rate is 35 kHz. Figure 10c indicates that the proposed method has a low harmonic distortion of ~25 dB at the first harmonic frequency. The result achieved by Wang’s method has a lower noise level than that of the envelope method because it has a better standard deviation. Besides, the signal bandwidth of the proposed method is narrowest of three methods.

Next, a stability experiment is conducted and experimental results are shown in Figure 10d. The MD is ~23 μm and the MD voltage is sampled in 30 min. The peak-to-peak MD voltage is ~0.015 V which is corresponding to ~26.76 nm for the proposed method. The tendency of Wang’s method is close to the proposed method but has many jumping errors. However, the tendency of the envelope method is slightly different from those of the proposed and Wang’s methods due to the influence of dispersion. The stability of the envelope method is worst and it is ~0.075 V which is corresponding to ~133.80 nm.

Finally, the repeatability at different MDs of 22.90 μm, 23.25 μm, 23.50 μm and 23.85 μm is tested. The experimental results are illustrated in Table 2. During the experiments, the PZT conducts zero returning and then goes back to the specified MDs. Meanwhile, MD voltage is sampled and recorded. Each experiment is conducted ten times. Experimental results indicate that the repeatability at different MDs is better than 8.39 nm.

## 4. Conclusions

In this paper, a MADM method of high accuracy and frequency response based on PLCI is proposed. This method gets rid of the dependences on the a priori model and birefringence parameters which makes it more robust and adaptable. The nearly linear relationship between envelope and the zero-order PLCIF centers of a Gauss and non-Gauss profile BLS is investigated in theoretical derivation, simulation and experimental methods. Then, polynomial fitting is found to be effective to compensate the nonlinearity induced by BOW’s dispersion and enhance the measurement accuracy. Compared to conventional methods, the proposed method is very robust and can effectively avoid jump errors. The experimental results indicate that the measurement accuracy is higher than 19.5 nm, the resolution is better than 2 nm, the repeatability at different MDs is better than 8.39 nm, the stability within 30 min is better than ~26.76 nm, the signal processing time on an embedded system is ~22 μs, its processing data rate can reach 35 kHz and the harmonic distortion is low than 25 dB under 1 kHz frequency response test. This method can achieve a high accuracy and frequency response for MADM. It has a promising application in the fields of FP sensor demodulation, vibration sensing and surface profile measurement.

## Figures and Tables

**Table 1:** The PLCIF with a BLS (broadband light source) in different profiles and relationship between *h_m_* and *h_c_*.

BLS Profile	*p* Parameter	*q* Parameter	Linear Fitting Residual/mm	Expression of PLCIF
Guass	1.0436	−0.1424	0.093	$\frac{R\sqrt{\pi }}{\alpha }\cdot \left\{1-\exp\left[-\frac{{\left(z\Delta k\right)}^{2}}{\gamma }\right]\cdot \cos\left[n_{0}k_{0}h-2k_{0}d\right]\right\}$
Square	1.0457	−0.1416	0.126	$\frac{2R\sin(\Delta kz/2)}{z}\cos\left[k_{0}z-hk_{0}^{2}\beta \right]$
Triangular	1.0461	1.0461	0.101	$\begin{array}{l} \frac{2R}{z}\{\left[1-\cos(\Delta kz/2)\right][\frac{1}{z}\cos(k_{0}z-hk_{0}^{2}\beta )-\frac{1}{\Delta k}\\ -1]+(\frac{1}{\Delta k}+1)\sin(\Delta kz/2)\cos\left[k_{0}z-hk_{0}^{2}\beta \right]\} \end{array}$
Measured	1.0450	−0.1380	0.141	none

**Table 2:** Experimental results of repeatability tests.

MD/μm	Measured MD Voltage/V					STD/mV	STD/nm
22.90	2.1324	2.1310	2.1304	2.1290	2.1276	3.8	6.79
	2.1259	2.1251	2.1247	2.1228	2.1204		
23.25	2.3054	2.3035	2.3031	2.3011	2.3004	3.9	6.96
	2.2991	2.2980	2.2960	2.2957	2.2931		
23.50	2.4777	2.4761	2.4747	2.4737	2.4729	3.6	6.43
	2.4716	2.4705	2.4697	2.4685	2.4658		
23.85	2.6815	2.6809	2.6808	2.6774	2.6765	4.7	8.39
	2.6729	2.6743	2.6705	2.6723	2.6677		
